# Role of atrial natriuretic peptide in the dissociation between flow relations with ventricular mass and function in a community with volume-dependent hypertension

**DOI:** 10.3389/fcvm.2023.1175145

**Published:** 2023-05-17

**Authors:** Suraj M. Yusuf, Gavin R. Norton, Vernice R. Peterson, Nonhlanhla Mthembu, Carlos D. Libhaber, Grace Tade, Hamza Bello, Adamu J. Bamaiyi, Keneilwe N. Mmopi, Patrick H. Dessein, Ferande Peters, Pinhas Sareli, Angela J. Woodiwiss

**Affiliations:** Cardiovascular Pathophysiology and Genomics Research Unit, School of Physiology, Faculty of Health Sciences, University of the Witwatersrand, Johannesburg, South Africa

**Keywords:** atrial natriuretic peptide, stroke volume, left ventricular mass, diastolic function, systolic function

## Abstract

**Background:**

Whether differential effects of volume load on left ventricular mass (LVM) and function occur in sustained volume-dependent primary hypertension, and the impact of atrial natriuretic peptide (ANP) on these effects, is unknown.

**Methods:**

From aortic pressure, velocity and diameter measurements and echocardiography, we determined in an African community (*n* = 772), the impact of systemic flow-induced increases in central pulse pressure (PPc) and circulating ANP (ELISA) on LVM and indexes of function.

**Results:**

Stroke volume (SV), but not aortic flow (Q), was associated with LVM and mean wall thickness (MWT) beyond stroke work and confounders (*p* < 0.0001). Adjustments for SV markedly decreased the relationships between PPc and LVMI or MWT. However, neither SV, nor Q were independently associated with either myocardial s', e', or E/e' (*p* > 0.14) and adjustments for neither SV nor Q modified relationships between PPc and s', e' or E/e' (*p* < 0.005 to <0.0001). SV was nevertheless strongly and independently associated with ANP (*p* < 0.0001) and ANP was similarly strikingly associated with s' (*p* < 0.0001) and e' (*p* < 0.0005), but not E/e', independent of confounders and several determinants of afterload. Importantly, ANP concentrations were inversely rather than positively associated with LV diastolic dysfunction (DD) (*p* < 0.005) and lower rather than higher ANP concentrations contributed markedly to the ability to detect DD in those with, but not without LV hypertrophy.

**Conclusion:**

In populations with sustained volume-dependent hypertension, flow (SV)-related increases in PP have a major impact on LV structure, but not on function, an effect attributed to parallel striking beneficial actions of ANP on myocardial function.

## Introduction

Conventional thought is that sustained increases in blood pressure (BP) in primary hypertension occur principally because of increases in vascular resistance to flow, rather than to an enhanced flow *per se*. Indeed, earlier community-based studies demonstrate increases in systemic flow in hypertensive individuals only at a younger adult age (1, 2). However, studies that are more contemporary show a marked contribution across the adult age range of increases in stroke volume (SV) and peak aortic flow (Q) to primary hypertension in African populations derived from equatorial regions (3–6). Consistent with genetic adaptations to hot, arid environments (7), these flow-dependent mechanisms are strongly associated with renal tubular changes (5) and demonstrate striking heritability estimates (8). Although current therapy, including therapy with diuretic agents, has no proven ability to produce sustained reductions in circulating volume (9), combinations of agents can achieve BP control over short-term periods in groups living in Africa (10, 11). Thus, whether volume-reduction is a necessary approach to managing BP in volume-dependent primary hypertension is unknown. In this regard, researchers have recognised a dissociation between the adverse cardiac effects of pressure and volume overload for over a half century.

Hypertrophy of the heart in pressure and volume overload states has consistently been demonstrated to produce differential effects on not only ventricular geometry (concentric vs. eccentric hypertrophy), but also on the extent of myocardial cellular abnormalities and dysfunction (12–16). Importantly, despite a similar degree of cardiac hypertrophy, pressure overload states generally produce adverse myocardial cellular and interstitial changes and dysfunction more consistently than do volume overload states (14, 15). However, most studies conducted with models of volume overload have employed arteriovenous fistulae or aortic regurgitation (12–14) where the volume overload does not translate into a combined volume and pressure load. In contrast, in populations with sustained volume-dependent primary hypertension, a combined pressure and volume overload occurs (3–6). In this regard, in contrast to current notions of the determinants of left ventricular (LV) geometry, in populations with sustained volume-dependent primary hypertension, concentric LV geometry is determined as much by increased volume and pressure as is eccentric LV geometry (17). Consistent with these data are preclinical data where the sequential addition of pressure overload to volume overload produced the same changes in cardiac myocyte diameter and length, as did the addition of volume overload to pressure overload (16). Although the degree of the pressure load is strongly associated with LV dysfunction (18), whether the volume load produces similar effects on LV mass (LVM) and LV function in sustained volume-dependent hypertension, is unclear. In volume-dependent populations, circulating atrial natriuretic peptide (ANP) concentrations are enhanced (6), and preclinical (19, 20) and clinical (21) studies indicate that ANP augments myocardial systolic and diastolic function, particularly in the presence of LV hypertrophy (19). Thus, it is possible that despite an adverse effect of volume load on pulse pressure (PP) and hence LVM, the increased release of ANP in volume overload states protects the LV from volume-induced effects on myocardial function. If so, targeting the volume overload state may not be a necessary requirement to prevent the transition to heart failure in sustained volume-dependent hypertension. To address this question in the present study conducted in a population with prevalent volume-dependent hypertension, we therefore evaluated whether volume-induced increases in PP produce differential effects on LVM and tissue Doppler indexes of myocardial function. We further assessed whether relationships between the enhanced ANP concentrations associated with the volume overload state (6) and myocardial function, in-part account for any dissociation between the impact of volume-dependent effects on LVM and function.

## Methods

### Study groups

The present study was conducted according to the principles outlined in the Helsinki declaration. The Committee for Research on Human Subjects of the University of the Witwatersrand approved the protocol (approval numbers: M02-04-72 and renewed as M07-04-69, M12-04-108, M17-04-01 and M22-03-93). Participants gave informed, written consent. The present study design has previously been described (3–6, 15, 18, 22, 23). Briefly, in a cross-sectional community-based study, nuclear families of black African descent (Nguni and Sotho chiefdoms) with siblings older than 16 years of age were randomly recruited (population census figures of 2001) from the South West Township (SOWETO) of Johannesburg, South Africa. In the present sub-study, 772 participants had high quality aortic velocity measurements in the outflow tract and ANP measurements, 537 of which had myocardial tissue Doppler imaging measurements.

### Clinical, demographic and blood data

A questionnaire was administered to obtain demographic and clinical data (23). Height and weight were measured using standard approaches and participants were considered to be overweight if their body mass index (BMI) was ≥25 kg/m^2^ and obese if their BMI was ≥30 kg/m^2^. Laboratory blood tests of renal function, liver function, blood glucose, lipid profiles, hematological parameters, and percentage glycated hemoglobin (HbA1c) were performed. Diabetes mellitus (DM) was defined as the use of insulin or oral glucose lowering agents or an HbA1c value greater than 6.5%. High quality office brachial BP measurements were obtained in the seated position and after 5 min of rest, by a trained nurse-technician using a standard mercury sphygmomanometer (23) according to guidelines. The mean of 5 measurements obtained at least 30 s apart was taken as office BP. Hypertension was defined as a mean office BP ≥ 140 mm Hg systolic or ≥90 mm Hg diastolic or the use of antihypertensive medication. Plasma concentrations of ANP were measured using an enzyme-linked immunosorbent assay (Elabscience Biotechnology Inc, Houston, Texas, USA) as previously described (6). Although, NTproANP is more stable and has a longer half-life than ANP, it has no known biological actions. Hence, we chose to measure the bioactive peptide circulating ANP.

### Hemodynamic assessments

Hemodynamics were determined non-invasively from central arterial pressure recordings obtained using pulse wave analysis together with aortic velocity and diameter assessments obtained in the outflow tract as previously described (3–6, 17, 22). After participants had rested for 15 min in the supine position, arterial waveforms at the radial (dominant arm) pulse were recorded by applanation tonometry using a high-fidelity SPC-301 micromanometer (Millar Instrument, Inc., Houston, Texas) interfaced with a computer employing SphygmoCor, version 9.0 software (AtCor Medical Pty. Ltd., West Ryde, New South Wales, Australia). A central arterial pulse was derived from the radial pulse using a validated generalized transfer function in SphygmoCor software. Immediately after central arterial pressure waveforms were obtained, an experienced observer (AJW) obtained aortic velocity and diameter measurements in the left lateral decubitus position using an Acuson SC2000 Diagnostic ultrasound system (Siemens Medical Solutions, USA, Inc.). Velocity waveforms were obtained in the 5-chamber view. High quality velocity assessments were identified as those with a smooth velocity waveform with a dense leading (outer) edge and a clear maximum velocity. Aortic diameter measurements were obtained just proximal to the aortic leaflets in the long axis parasternal view. Peak aortic flow (Q) was determined as the product of peak aortic velocity and aortic cross-sectional area (determined from diameter measurements). Stroke volume (SV) was calculated from the product of the velocity-time integral and aortic root cross-sectional area. Stroke volume was also determined from the difference between end diastolic and end systolic volumes calculated using the biplane Simpson approach in a 4-chamber view (3). Cardiac output (CO) was calculated from SV x heart rate (HR). Systemic vascular resistance (SVR) was calculated from mean arterial pressure (MAP)/CO, assuming that right atrial pressure = 0 mm Hg, and where MAP was determined from the arterial pressure wave using SphygmoCor software (3). To assess the extent that relationships between SV and LV structure were independent of workload, relationships were adjusted for stroke work (SW), where SW was determined from the product of SV and peak central arterial systolic BP generated during ventricular ejection x 0.014 (24).

To assess the relationships between ANP and LV function independent of pulsatile loading conditions, adjustments for several indexes of pulsatile load were performed. Pulsatile load was assessed from characteristic impedance to flow (Zc), forward (Pf) and backward (Pb) wave pressures, and the pressures generated by the product of peak aortic flow (Q) and Zc (P_QxZc_). In line with the recommendation that either time or frequency domains may be employed for the assessment of Zc (25), Zc was determined in the time domain using approaches previously described by groups from both Framingham and Ghent (26, 27) and validated against invasive pressure measurements (28). The volume flow waveform was paired with central arterial pressure waveforms by aligning the foot (t_0_) of the respective signal averaged waveforms. The point at which flow achieves 95% of its peak (t_Q95_) was identified. The corresponding pressure change between t_0_ and t_Q95_ was determined. Characteristic impedance was calculated as the ratio of change in pressure to change in flow in the window t_0_ to t_95_. Using Zc values and flow and pressure waveforms, wave separation analysis was performed and Pf determined from (aortic PP + QxZc)/2 and Pb from (aortic PP—QxZc)/2 (3). The contribution to Pf of pressures determined by an interaction between Q and Zc was identified from the pressures generated by the product of peak aortic Q and Zc (P_QxZc_) (29).

### Left ventricular mass and function

Echocardiographic measurements were performed as previously described (17, 18, 23) by two experienced observers (AJW and CDL) with a low degree of intra- and inter-observer variability, with the participants in the partial left decubitus position. Left ventricular dimensions were determined using two-dimensional directed M-mode echocardiography in the short axis view and these recordings were analyzed according to the American Society of Echocardiography convention (30). Left ventricular mass (LVM) was determined from end diastolic septal and posterior wall thickness and internal diameters using a standard formula (31) and indexed to height to the allometric signal of 1.7 (LVM index, LVMI) (17). Left ventricular mean wall thickness (MWT) was determined as the mean of septal and posterior wall thickness at end diastole. Left ventricular diastolic function was assessed using transmitral velocity and tissue Doppler imaging as previously described (17, 18) and data are shown as the mean velocity of myocardial lengthening in early diastole (e') in the lateral and septal walls and transmitral velocity in early diastole (E)/e', an index of LV filling pressure. Left ventricular systolic function was assessed using tissue Doppler imaging as previously described (17) and data are shown as the mean velocity of myocardial shortening in the lateral and septal walls (s'). The presence of LV diastolic dysfunction (LV DD) was determined as previously described (18), based on criteria which include a reduced myocardial e' in the septum or lateral wall, an increased E/e' and an enhanced left atrial volume.

### Data analysis

SAS software, version 9.4 (SAS Institute Inc., Cary, NC) was used for database management and statistical analysis. Continuous variables are expressed as mean (± SD or SEM). Dichotomous variables are expressed as percentages. To identify independent relationships, multivariate adjusted linear regression analysis was performed. Adjustments were for age, sex, MAP, regular alcohol intake, regular tobacco intake, BMI, diabetes mellitus, treatment for hypertension, heart rate and additional hemodynamic factors as indicated. As treatment for hypertension may affect relationships, sensitivity (secondary) analysis was conducted in untreated participants. As more women than men volunteered for the study, sensitivity analysis was also conducted in women and men separately.

## Results

### Participant characteristics

Table 1 shows the characteristics of the study sample. A high proportion of participants had hypertension and obesity, and a significant proportion had uncontrolled BP values despite a number of these individuals receiving antihypertensive medication. Of the sample 39.9% had LVH (LVMI > 60 g/m^1.7^ for women and >80 g/m^1.7^ for men) and 16.0% had LV DD. A greater proportion of participants with (28.5%) as compared to without (7.7%) LVH had LV DD.

**Table 1:** Characteristics of community sample.

Sample size (% women)	772 (67.9)
Age (years)	46.3 ± 18.1
Body mass index (kg/m^2^)	29.7 ± 7.7
%Overweight/%obese	25.9/44.0
Hypertensive (%)	47.3
Treated hypertensives (%)	26.9
% with normal BP (<140/90 mm Hg)	64.6
Diabetes mellitus (%)	13.3
Regular smoking (%)	15.3
Regular alcohol (%)	19.7
Systolic blood pressure (mm Hg)	128 ± 22
Diastolic blood pressure (mm Hg)	83 ± 12
Mean arterial pressure (MAP) (mm Hg)	99 ± 15
Central arterial pulse pressure (PPc)	35 ± 14
Heart rate (beats/min)	68 ± 12
Stroke volume (SV) (outflow tract) (mls/beat)	81.4 ± 35.5
Peak aortic flow (Q)(mls/sec)	350 ± 165
SV (outflow tract) (mls/beat.BSA)	45.5 ± 19.6
SV (LV dimensions) (mls/beat)	81.9 ± 27.8
SVR (outflow tract) (mm Hg.min/litre)	20.7 ± 9.0
SVR (LV dimensions) (mm Hg.min/litre)	20.4 ± 8.3
Characteristic impedance (Zc) (dynes.cm^−5^)	85.3 ± 44.1
Forward wave pressure (Pf)(mm Hg)	27 ± 9
Backward wave pressure (Pb)(mm Hg)	13 ± 6
P_QxZc_ (mm Hg)	26 ± 8
Left ventricular mass index (LVMI-ht^1.7^)(g/m^1.7^)	64.8 ± 22.7
LV mean wall thickness (cm)	0.85 ± 0.17
Mean LV wall s’ (cm/s) (*n* = 537)	8.9 ± 2.7
Mean LV wall e’ (cm/s) (*n* = 537)	11.3 ± 4.2
LV E/e’ (*n* = 537)	7.5 ± 4.3
Atrial natriuretic peptide (ANP) (pg/ml)	24.5 (16.6 to 41.4)*

Outflow tract, data calculated from measurements in the left ventricular outflow tract; LV dimensions, data calculated from measurements of left ventricular dimensions; SVR, systemic vascular resistance; P_QxZc_, peak of the pressure wave generated by the product of Q and Zc; s’, velocity of LV myocardial tissue shortening at the level of the mitral annulus; e’, velocity of early diastolic LV myocardial tissue lengthening at the level of the mitral annulus; E, velocity of early diastolic trans-mitral blood flow; E/e’, an index of LV filling pressure.

^*^ Data are shown as mean ± SD, proportions or median and interquartile range.

### Systemic flow and BP

Independent of confounders, both peak aortic flow (Q) and stroke volume (SV) were strongly and independently associated with all pressure assessments, including central arterial SBP (SBPc), or PP (PPc) and MAP, Pf, and P_QxZc_ (Supplementary Table S1). Importantly, relationships between SV or Q and SBP or PP were independent of each other (*p* < 0.0005 for both SV and Q).

### Dissociation between the impact of systemic flow-induced increases in PPc on LV structure and function

**Figure 1:**
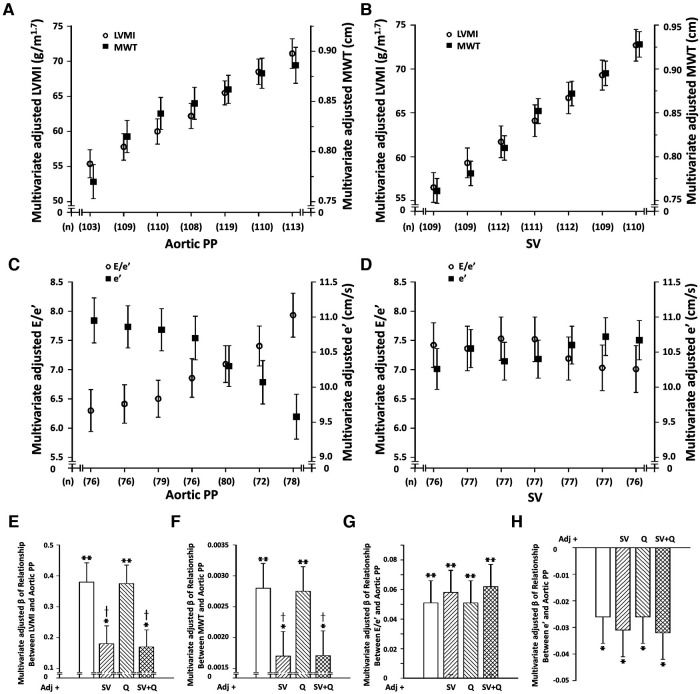
Dissociation of independent relationships between volume-dependent (stroke volume [SV] or peak aortic flow [Q]) increases in central arterial pulse pressure (PPc) and left ventricular (LV) structure and function in a community with prevalent volume-dependent hypertension. (Panels **A–D**) show multivariate adjusted LV structure and function across septiles of SV or PP and panels (**E-H**) show impact of adjustments for SV or Q on PPc-LV structure or function relations. LVMI, LV mass index; MWT, LV mean wall thickness; e’, early diastolic mean LV myocardial tissue lengthening velocity at the level of the mitral annulus; E, early diastolic trans-mitral blood flow velocity; E/e’, index of LV filling pressure. Adjustments are for age, sex, MAP, regular alcohol intake, regular tobacco intake, BMI, diabetes mellitus, treatment for hypertension, heart rate, and hemodynamic factor as indicated. See Supplementary Figure S1 for s’ effects.

Central arterial PP (PPc) was strongly and independently associated with LVMI and MWT (Figure 1 and Supplementary Tables S2–S4), as well as LV diastolic (decreased myocardial e' and increased E/e’) (Figure 1 and Supplementary Tables S2–S4) and systolic (decreased myocardial s') (Supplementary Figure S1 and Supplementary Tables S2–S4) function. In contrast, while SV (Figure 1 and Supplementary Tables S2–S4), but not Q was strongly and independently associated with both LVMI and MWT, neither SV (Figures 1 and Supplementary Figure S1), nor Q were independently associated with LV diastolic or systolic function. Importantly, the relationship between SV and LVMI was independent of both Q and SW (Supplementary Table S5). Although SW showed independent relationships with LVMI beyond SV and Q, SV, but neither Q nor SW showed independent associations with MWT (Supplementary Table S5). In addition, further adjustments for SV, but not Q markedly attenuated relationships between PPc and either LVMI or MWT (Figures 1E,F and Supplementary Tables S2–S4), while further adjustments for neither SV nor Q modified the independent relationships between PPc and either LV diastolic (Figures 1G,H and Supplementary Tables S2–S4) or systolic (Supplementary Figure S1) function.

### Relationships between ANP and LV function

**Figure 2:**
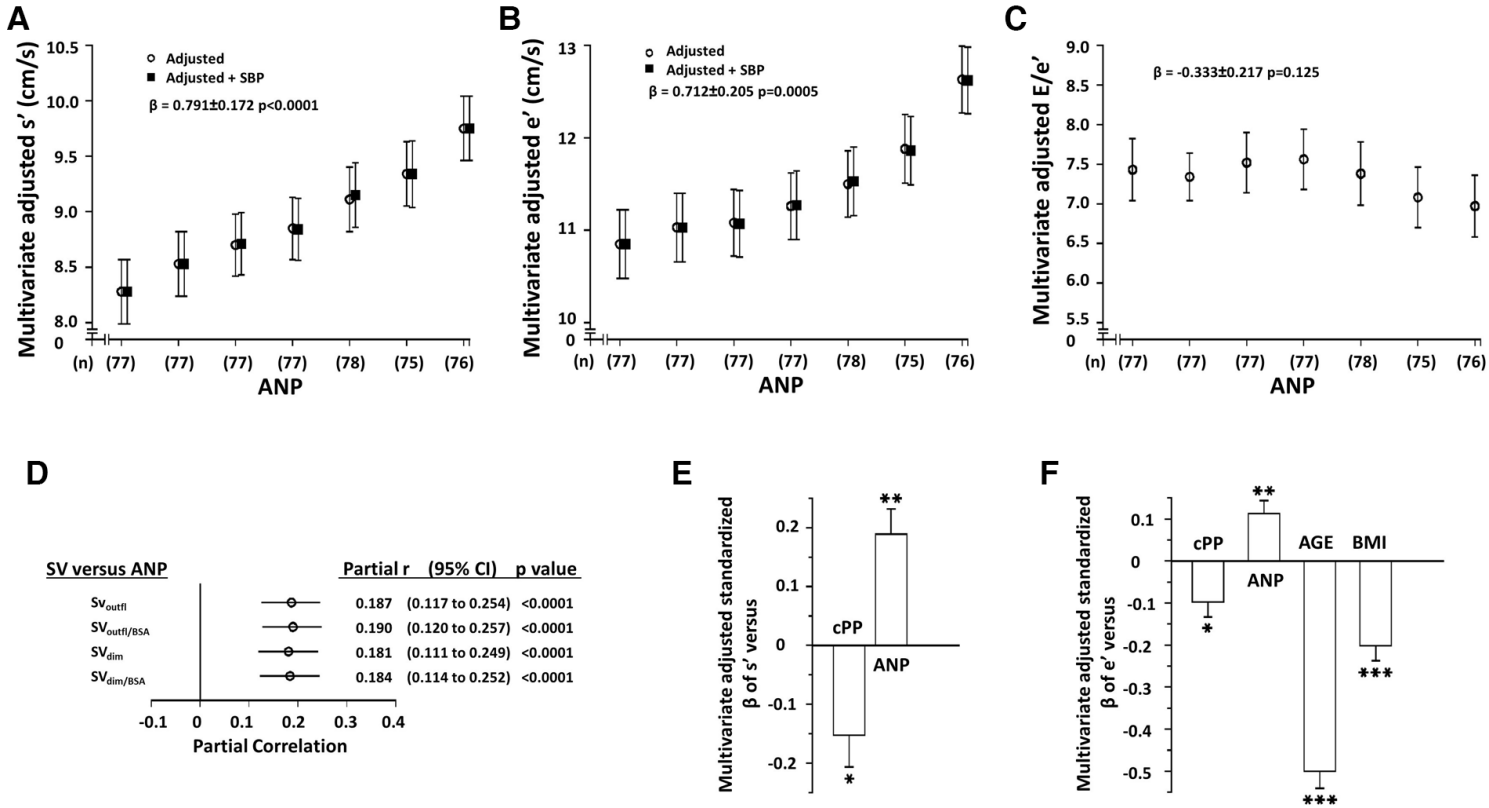
Independent relationships between circulating atrial natriuretic peptide concentrations (ANP) and left ventricular (LV) function (n= 537) (panels **A–C**) and between hemodynamic indexes of volume overload (stroke volume, SV) and ANP (panel **D**, *n* = 772) in a community with prevalent volume-dependent hypertension. Panels **A-C** show multivariate adjusted LV function across septiles of ANP, panel **D** shows the correlations between SV and SNP after adjustments for confounders (partial correlations), and panels **E** and **F** show the relative impact (standardized β-coefficients) of factors associated with LV function. SBPc, central arterial systolic blood pressure. See Figure 1 and Table 1 for other abbreviations. Adjustments are for age, sex, MAP, regular alcohol intake, regular tobacco intake, BMI, diabetes mellitus, treatment for hypertension, heart rate, and hemodynamic factor as indicated.

Independent of confounders, SV was independently associated with ANP concentrations irrespective of whether SV was derived from outflow tract measurements or LV dimensions and whether SV was indexed to BSA or not (Figure 2D). Circulating ANP concentrations were in turn strongly, independently, and directly associated with both myocardial s' and e', but not E/e' and these relationships were unaffected by adjustments for SBPc (Figures 2A–C, Table 2). Importantly, the beneficial relationships between ANP concentrations and myocardial function were also independent of several determinants of afterload to the LV including PPc and MAP, SVR, Zc, Pf and Pb, and P_QxZc_ (Tables 3 and Supplementary Tables S6–S8). In regression models, the magnitude (standardised *β*-coefficient) of the positive impact of ANP concentrations on myocardial s' and e' was at least as strong as that of the inverse effect of PPc on myocardial s' and e' (Figures 2E,F) and the strength of the effect on e' was third only to age and BMI (Figures 2E,F). Although ANP concentrations were strongly associated with hemodynamic indexes of volume load (Figure 2D), the impact of ANP on myocardial function translated into reduced, rather than increased, ANP concentrations in those with LV diastolic dysfunction (LV DD) (ANP in pg/ml, No DD = 35.3 ± 1.3, DD = 25.6 ± 3.1, *p* < 0.005). Further, lower rather than higher ANP concentrations were independently associated with LV DD (Supplementary Table S9). Importantly the magnitude (standardised β-coefficient) of the contribution of ANP concentrations to the ability to detect LV DD was similar to that of age and PPc (Supplementary Table S9).

**Table 2:** Independent relationships between atrial natriuretic peptide concentrations (ANP) and left ventricular function in participants not receiving antihypertensive therapy (untreated) and in women and men of a community with prevalent volume-dependent hypertension.

ANP versus	β-coefficient ± SEM	*p* value
	Untreated (*n* = 387)	
Myocardial s’	0.865 ± 0.202	<0.0,001
Myocardial e’	0.682 ± 0.245	<0.01
Myocardial E/e’	−0.290 ± 0.220	=0.19
	Women (*n* = 365)	
Myocardial s’	0.870 ± 0.214	<0.0,001
Myocardial e’	0.661 ± 0.245	<0.01
Myocardial E/e’	−0.349 ± 0.279	=0.21
	Men (*n* = 172)	
Myocardial s’	0.824 ± 0.295	<0.01
Myocardial e’	0.783 ± 0.354	<0.05
Myocardial E/e’	−0.261 ± 0.333	=0.44

See Figure 1 and Table 1 for other abbreviations. Adjustments are for age, sex (except in sex-specific groups), MAP, regular alcohol intake, regular tobacco intake, BMI, diabetes mellitus, treatment for hypertension (except in untreated), and heart rate.

**Table 3:** Impact of adjustments of determinants of left ventricular afterload on independent relationships between atrial natriuretic peptide concentrations (ANP) and left ventricular function in a community with prevalent volume-dependent hypertension (*n* = 537).

ANP versus	Adjustments	β-coefficient ± SEM	*p* value
Myocardial s’	*	0.796 ± 0.179	<0.0,001
	* + PP	0.826 ± 0.180	<0.0,001
	* + SVR	0.791 ± 0.180	<0.0,001
	* + Zc	0.781 ± 0.181	<0.0,001
	* + Pf and Pb	0.822 ± 0.181	<0.0,001
	* + P_QxZc_	0.823 ± 0.181	<0.0,001
Myocardial e’	*	0.719 ± 0.203	<0.0,005
	* + PP	0.712 ± 0.205	<0.0,005
	* + SVR	0.681 ± 0.204	<0.001
	* + Zc	0.709 ± 0.204	<0.0,005
	* + Pf and Pb	0.727 ± 0.201	<0.0,005
	* + P_QxZc_	0.715 ± 0.203	=0.0,005
Myocardial E/e’	*	−0.261 ± 0.223	=0.24
	* + PP	−0.333 ± 0.217	=0.13
	* + SVR	−0.297 ± 0.218	=0.17
	* + Zc	−0.253 ± 0.218	=0.25
	* + Pf and Pb	−0.271 ± 0.216	=0.21
	* + P_QxZc_	−0.255 ± 0.218	=0.24

See Figure 1 and Table 1 for other abbreviations. Adjustments are for age, sex, MAP, regular alcohol intake, regular tobacco intake, BMI, diabetes mellitus, treatment for hypertension, heart rate and hemodynamic factors as indicated.

Of note, strong independent relationships between myocardial s' and e' were noted (Supplementary Figure S2) and consequently, relationships between ANP concentrations and LV diastolic function (relaxation) (e') were markedly attenuated by adjustments for myocardial systolic function (s') (Supplementary Figure S3). In addition, as myocardial relaxation (e’) was so strongly determined by myocardial shortening (s'), LV filling pressures (E/e') were similarly strongly determined by myocardial s' (Supplementary Figure S2).

### Relationships between ANP and LV function in LVH

**Figure 3:**
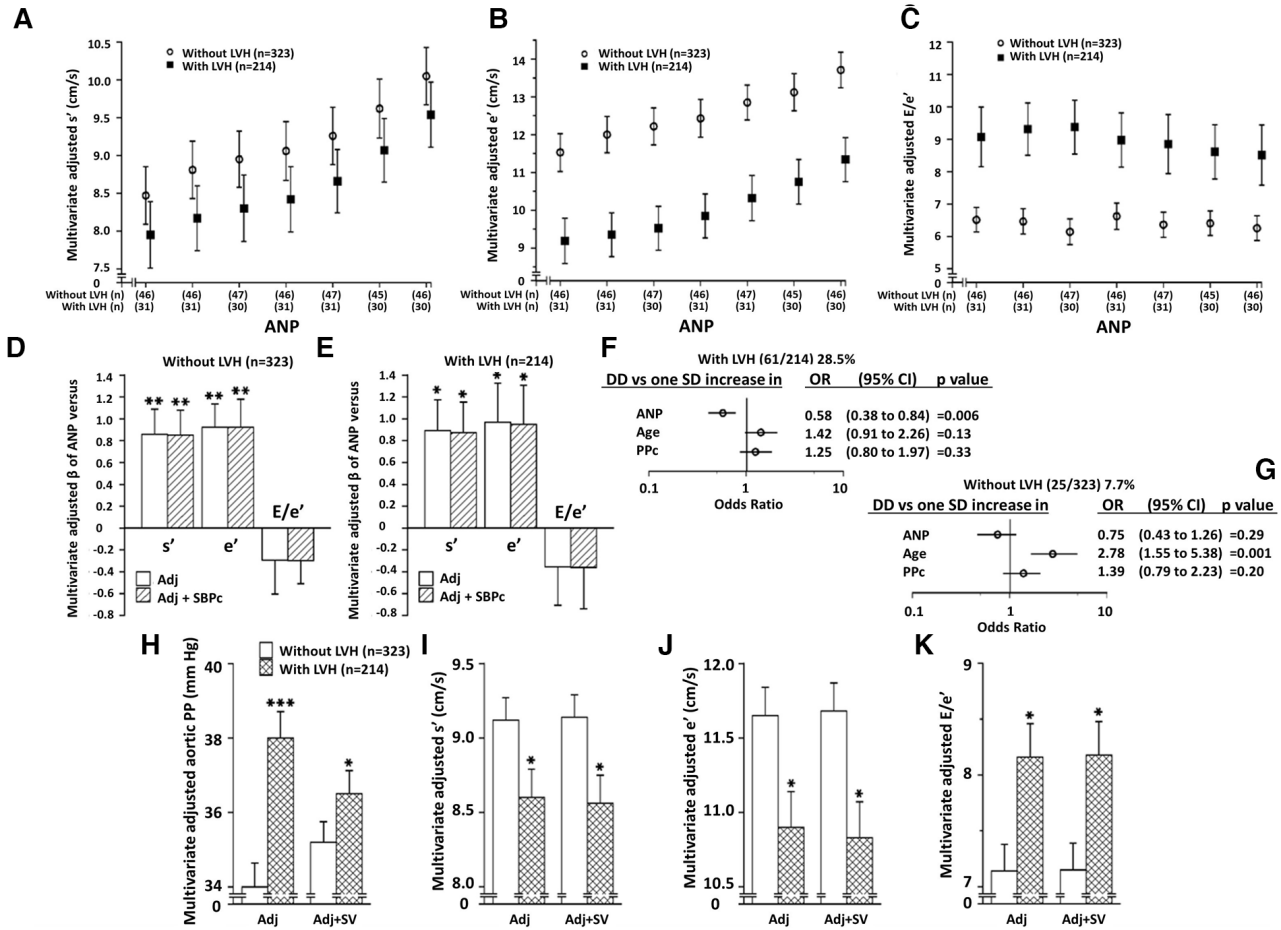
Independent relationships between circulating atrial natriuretic peptide concentrations (ANP) and left ventricular (LV) function in those with and without LV hypertrophy (LVH). Panela **A–C** show multivariate adjusted LV function across septiles of ANP in those with and without LVH. Panels **D** and **E** show the impact of adjustments for BP on ANP-LV function relations in those with and without LVH, and panels **F**,**G** show the contribution of ANP versus age and PPc to LV diastolic dysfunction (DD) in those with and without LVH. Panels **H-K** show the impact of adjustment for volume overload [stroke volume (SV)] on PPc and LV function in those with and without LVH. See Figure 1 and Table 1 for other abbreviations. Adjustments are for age, sex, MAP, regular alcohol intake, regular tobacco intake, BMI, diabetes mellitus, treatment for hypertension, heart rate, and hemodynamic factor as indicated.

The independent relationships between ANP concentrations and myocardial s' and e' were noted in participants with or without LVH (Figures 3A,B). Adjustments for central arterial SBP failed to modify these relationships in either group (Figures 3D,E). No differences in the magnitude of these relationships were noted between those with and without LVH (Figures 3D,E). However, as those with LVH had markedly lower myocardial e' and higher E/e' values compared to those without LVH (Figures 3B,C), ANP concentrations, but neither age nor PPc had a major impact on the ability to detect those with LV DD in those with, but not without LVH (Figures 3F,G). In this regard, as with the whole group (Supplementary Table S9), lower and not higher ANP concentrations were independently associated with LV DD in those with LVH (Figure 3F). Importantly, while adjustments for SV markedly attenuated differences in PPc between those with and without LVH, adjustments for SV failed to modify differences in myocardial function between those with and without LVH (Figures 3H–K).

## Discussion

The main findings of the present study are as follows: In a population with prevalent volume-dependent hypertension (3–6), volume overload (SV and Q) was strongly associated with increases in BP, and SV-induced increases in PP accounted for a significant proportion of variations in LVMI and mean wall thickness (MWT). However, SV-related increases in PP failed to account for variations in tissue Doppler indexes of myocardial systolic or diastolic function. This was despite the marked contribution of PP to variations in both LV structure and function. Parallel volume-induced increases in ANP concentrations and the striking independent ability of ANP to associate with enhanced myocardial systolic and diastolic function, explained the lack of effect of SV-induced increases in PP on LV function. Importantly, ANP concentrations were positively associated with LV systolic and diastolic function and hence lower rather than higher ANP concentrations were independently associated with LV diastolic dysfunction. Lower rather than higher ANP concentrations thus markedly enhanced the ability to detect LV diastolic dysfunction in those with, but not without LVH.

While a dissociation between the impact of pressure and volume overload-induced cardiac hypertrophy on cardiac function has been well recognised for many years (12–14), no studies have identified whether a similar dissociation occurs when a pressure load is attributed in part to a volume load. This question has not received attention as pressure overload states, such as occur in hypertension, are thought to be primarily the result of increases in resistance or impedance to flow, rather than to increases in flow itself. Studies that are more contemporary nevertheless show that in specific populations, including those of African ancestry, a volume load is a fundamental cause of a pressure load (hypertension) across the full adult age range (3–6). In the present study we show for the first time that when a pressure overload state (indexed by PP) is in-part caused by a volume load [indexed by stroke volume (SV)], the volume-induced increase in PP, although markedly enhancing LVM and mean wall thickness, does not translate into LV dysfunction. We demonstrate that the parallel volume-induced increases in ANP, which strongly and independently associate with an improved myocardial function, even in those with LVH, explains the lack of impact of PP on LV function. In this regard, ANP showed a relationship with myocardial function with a magnitude second only to age and similar to PP for e' and with a similar effect on s' as PP. The impact of ANP on myocardial function thus markedly enhanced the ability to detect LV DD in those with LVH, with lower rather than higher ANP concentrations associating with LV DD. In this regard, through preload-induced effects on the LV, LV DD has traditionally been associated with increased and not decreased natriuretic peptide release from the myocardium.

Although, in the present community sample, ANP concentrations were independently associated with myocardial relaxation (myocardial e'), no relationships were noted with an index of LV filling pressure, E/e'. Yet, systemic flow-induced increases in PP were associated with neither myocardial relaxation, nor LV filling pressures, suggesting the presence of a protective mechanism against BP-induced effects on both myocardial relaxation and filling pressures. This apparent inconsistency may be explained by increases in filling volumes contributing to an enhanced LV filling pressure, while simultaneously promoting ANP release. In this regard, the ANP release is likely to attenuate the filling volume effect on filling pressures by increasing myocardial relaxation. Thus, ANP-induced decreases in filling pressures may oppose volume-induced increases in filling pressures, with neither filling volume nor ANP concentrations consequently showing independent relationships with LV filling pressures. Indeed, in the present study neither ANP concentrations (*p* = 0.13), nor filling volumes (end diastolic volume) (*p* = 0.54) were associated with E/e'.

Although several studies have demonstrated a role for ANP in enhancing myocardial systolic and diastolic function, both preclinical and clinical studies have provided controversial data (19, 21, 32). Studies reporting a lack of effect of ANP on myocardial function were frequently conducted in animal models or patients with heart failure, where alterations in second messenger systems in the myocardium modify the myocardial receptor response to ANP (19, 21, 32). However, as reviewed (19), the controversy in preclinical models was resolved by demonstrating an effect in models of pressure overload with, but not without LVH. In this regard, in the present study we nonetheless show robust independent relationships between ANP and myocardial systolic and diastolic function in both those with and without LVH. However, as those with LVH had markedly reduced LV diastolic function, ANP had a discriminating effect on the ability to detect LV DD in those with, but not without LVH. However, neither age nor PPc further enhanced the ability to detect DD. Importantly, ANP-myocardial function relationships were noted beyond all known determinants of afterload including PP, MAP, systemic vascular resistance, aortic characteristic impedance, and both forward and backward travelling pressure waves. Thus, the relationships between ANP and myocardial function noted in the present study cannot be attributed to the confounding beneficial effects of ANP on several vascular parameters previously described in the present population (6).

Although sustained volume-dependent hypertension is prevalent in the present community, several alternative vascular determinants of BP contribute to increases in BP (3–6). Thus, although there are no antihypertensive agents, including diuretic agents, which can produce persistent reductions in volume load (9), combinations of current agents, can produce significant BP reduction (10, 11). Thus, the question arises as to whether volume reduction in volume-dependent hypertension is necessary. In this regard, volume overload hypertrophy can produce myocardial dysfunction; however, it is less pronounced, or produced by different molecular pathways, than it is in pressure overload states (12–14). Importantly, we have previously demonstrated that age-related increases in backward wave pressures account for pressure wave effects on myocardial dysfunction in volume-dependent hypertension (18). Together with the present study which demonstrates a mechanism (increased ANP release) to protect myocardial function from the adverse impact of volume-induced PP effects, these studies suggest that targeting backward wave pressures and not volume overload is essential in preventing the transition from hypertension to heart failure in volume-dependent hypertension. In this regard, reductions in backward wave pressures can be achieved by decreasing forward wave pressures (backward wave pressures depend on forward wave pressures through Newton's Laws of motion). Aortic forward wave pressures can be reduced by decreasing aortic distending pressures (MAP), attenuating aortic characteristic impedance (Zc) and hence reducing the pressure wave produced by the product of peak aortic flow (Q) and Zc (the major component of the forward travelling pressure wave). The present study also suggests that enhancing the volume-dependent effect on circulating ANP concentrations in volume-dependent hypertension, using neprilysin inhibitors, may be a more effective approach to preventing the transition to heart failure in patients with hypertensive heart disease caused by volume-dependent hypertension. To address this point, further clinical studies are warranted.

There are several limitations to the present study. First, this was a cross-sectional study and hence the relationships described may not be cause and effect. However, intervention studies are not possible as presently no approaches reduce volume overload or decrease ANP concentrations in human volume-dependent primary hypertension. Currently, combined neprilysin inhibitors and angiotensin-II receptor antagonists approval is only for the treatment of heart failure and not for hypertension. However, in support of the present study, prospective studies (duration of 3 to 24 months) in patients with heart failure report increases in cardiac systolic function in response to combined neprilysin inhibitors and angiotensin-II receptor antagonists (33–36). Second, although when compared to volume overload hypertrophy, pressure overload hypertrophy produces adverse myocardial effects more consistently, volume overload can be sufficiently severe that it will lead to myocardial dysfunction (12–14). Whether the extent of volume overload in sustained volume-dependent primary hypertension ultimately produces these effects, is uncertain. However, in the present study conducted across the full adult age range, despite marked effects of SV on PP, SV showed no relationship with LV function. Third, the relationship between SV and LVMI may be attributed to an effect of stroke work (SW) (24), and hence the dissociation between SV effects on LV structure and function noted in the present study may be because LV structure is determined by workload and not by pressure effects. However, mean LV wall thickness was determined by SV and not by SW and we assessed the impact of SV on PPc-induced increases in both LVM and mean wall thickness. Fourth, as the present study was conducted in one ethnic group, our findings may therefore not apply to other ethnic groups.

In conclusion, in the present study we show that despite a striking effect of stroke volume-induced increases in PP on LVM and wall thickness in a volume-dependent population, volume-induced increases in PP do not cause LV dysfunction. We attribute this effect in part to a parallel release of ANP caused by the volume load, which has a significant beneficial action on myocardial function, the consequence being that decreased rather than increased ANP concentrations were independently associated with LV DD. These data suggest that targeting volume overload may not be an essential approach to preventing the transition to heart failure in volume-dependent primary hypertension. Nevertheless, these data also raise the question of whether enhancing circulating ANP concentrations with current antihypertensive agents (neprilysin inhibitors) may have added advantages in patients with hypertensive heart disease caused by volume-dependent hypertension.

## Data Availability

The raw data supporting the conclusions of this article will be made available by the authors, upon reasonable request.
