# Supplementary material for: Role of atrial natriuretic peptide in the dissociation between flow relations with ventricular mass and function in a community with volume-dependent hypertension
Source: Front Cardiovasc Med. 2023 May 17;10:1175145. doi: 10.3389/fcvm.2023.1175145 (PMC10230032; doi:10.3389/fcvm.2023.1175145)
Supplement: Supplementary file 1 [file Datasheet1.doc]

**
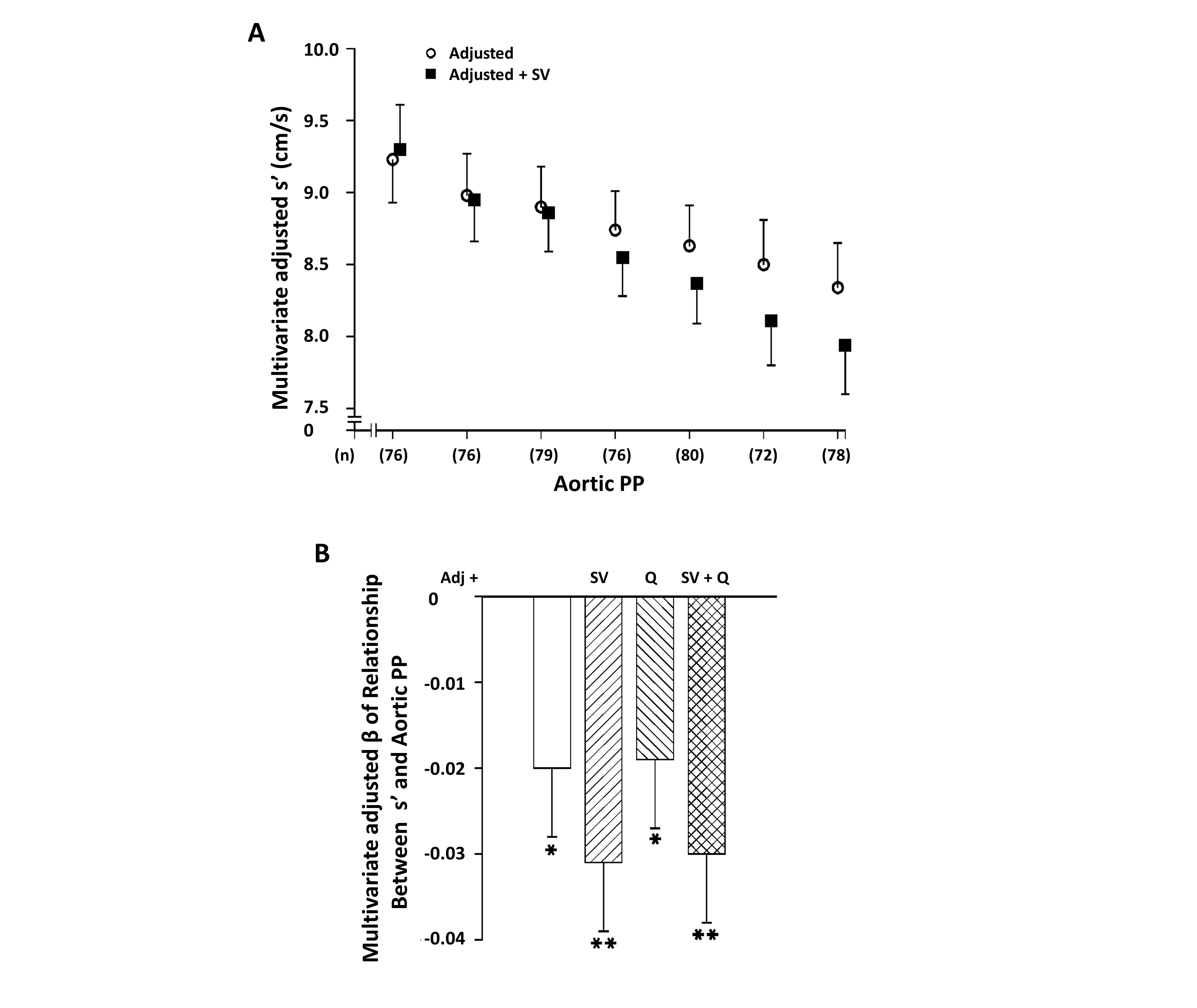
**

**Figure S1.** Impact of adjustments for volume overload (stroke volume [SV] or peak aortic flow [Q]) on relationships between central arterial pulse pressure (PPc) and left ventricular (LV) systolic function in a community with prevalent volume-dependent hypertension. Panel A shows multivariate adjusted LV systolic function across septiles of PPc before and after adjustments for SV and panel B shows impact of adjustments for SV or Q on PPc-LV systolic function relations. s’, velocity of myocardial tissue shortening at the level of the mitral annulus. Adjustments are for age, sex, MAP, regular alcohol intake, regular tobacco intake, BMI, diabetes mellitus, treatment for hypertension, heart rate, and hemodynamic factor as indicated.

**
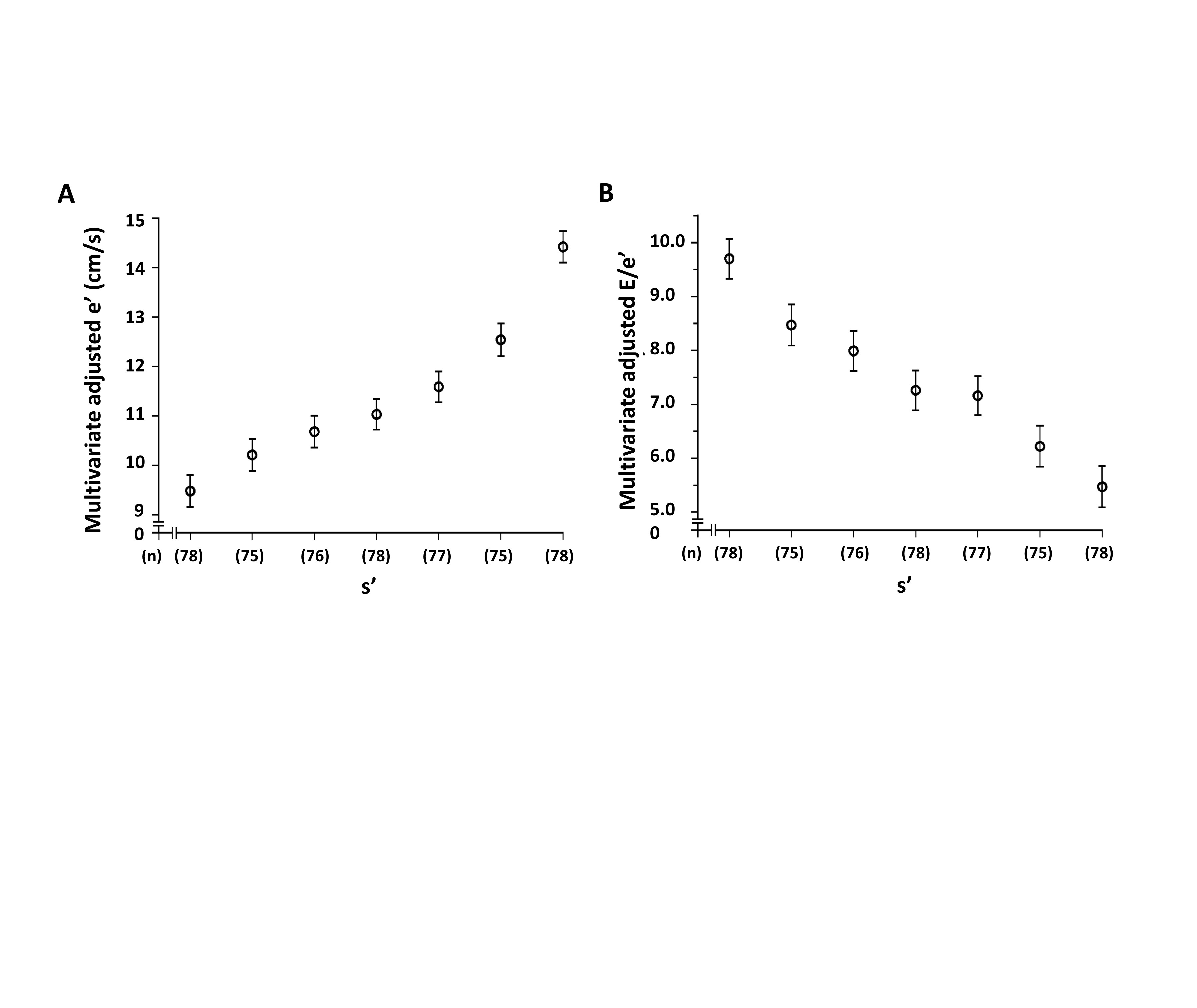
**

**Figure S2.** Independent relationships between indexes of systolic and diastolic function in a community with prevalent volume-dependent hypertension (n=537). See Table 1 for abbreviations. Adjustments are for age, sex, MAP, PPc, regular alcohol intake, regular tobacco intake, BMI, diabetes mellitus, treatment for hypertension, and heart rate.

**
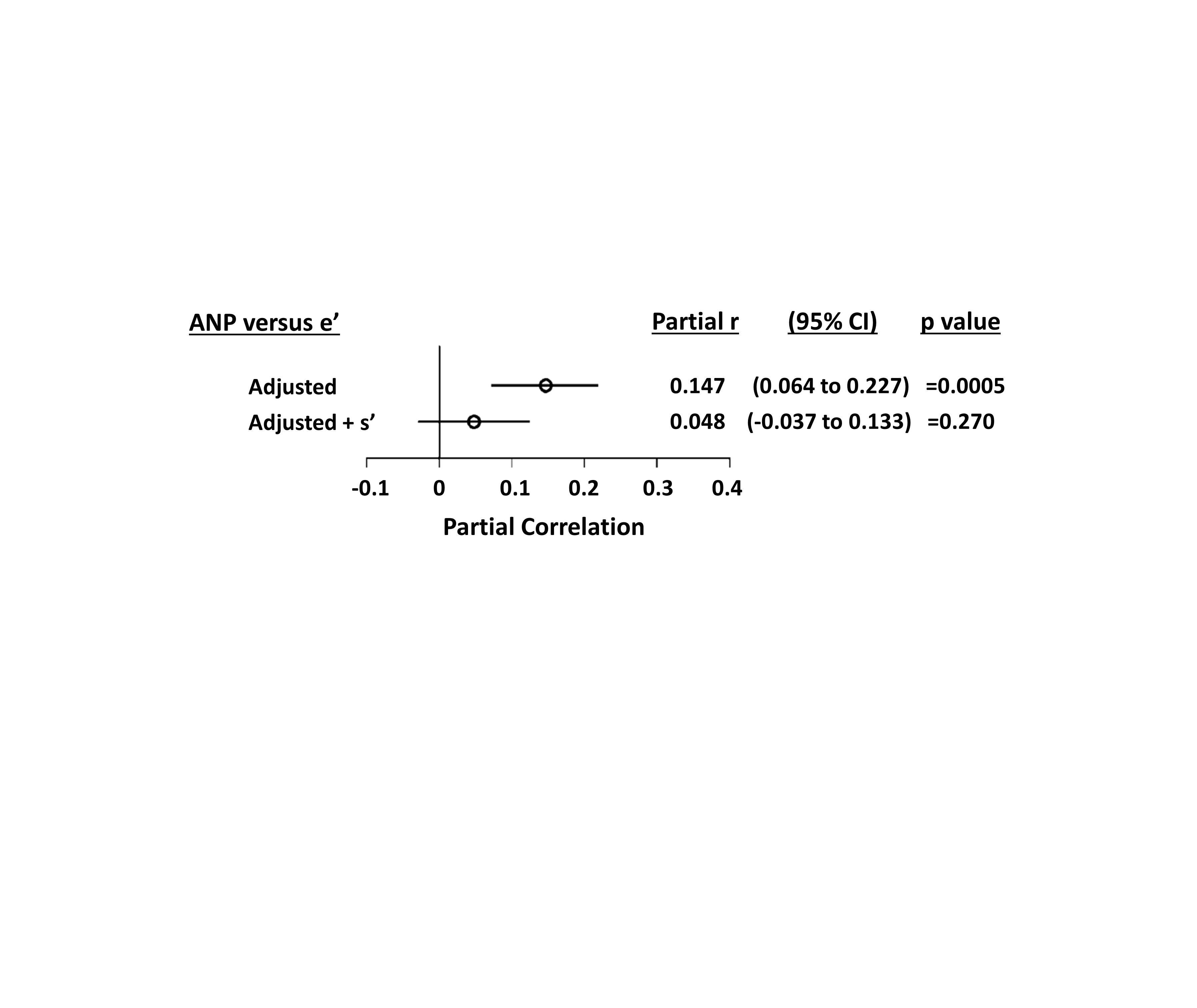
**

**Figure S3.** Impact of adjustments for myocardial systolic function (myocardial s’) on independent relationships between atrial natriuretic peptide concentrations (ANP) and myocardial relaxation (myocardial e’) (n=537). See Table 1 for abbreviations. Adjustments are for age, sex, MAP, PPc, regular alcohol intake, regular tobacco intake, BMI, diabetes mellitus, treatment for hypertension, and heart rate.

**Table S1**. Independent relationships between indexes of systemic flow and blood pressure or the determinants thereof in a community with prevalent volume-dependent hypertension (n=772).

β-coefficient±SEM p value

_________________________________________________________________

Stroke volume (SV) versus

Central systolic blood pressure (SBPc) 0.317±0.068 <0.0001

Central pulse pressure (PPc) 0.270±0.049 <0.0001

MAP 0.247±0.021 <0.0001

Forward wave pressures (Pf) 0.201±0.040 <0.0001

P_QxZc_ 0.195±0.039 <0.0001

Peak aortic flow (Q) versus

Central systolic blood pressure (SBPc) 0.269±0.031 <0.0001

Central pulse pressure (PPc) 0.181±0.035 <0.0001

MAP 0.170±0.028 <0.0001

Forward wave pressures (Pf) 0.247±0.026 <0.0001

P_QxZc_ 0.259±0.024 <0.0001

_______________________________________________________________

See Figure 1 and Table 1 for abbreviations. Adjustments are for age, sex, MAP (except for relations with MAP), regular alcohol intake, regular tobacco intake, BMI, diabetes mellitus, treatment for hypertension, and heart rate.

**Table S2**. Dissociation of relationships between volume (stroke volume [SV])-dependent increases in central arterial pulse pressure (PPc) and left ventricular (LV) structure and function in participants not receiving antihypertensive therapy (n=564).

Adjustments β-coefficient±SEM p value

____________________________________________________________

Stroke volume versus

LV mass index * 0.104±0.020 <0.0001

Mean LV wall thickness * 0.00151±0.00017 <0.0001

Myocardial s’ * 0.0056±0.0033 =0.094

Myocardial e’ * 0.0038±0.0034 =0.27

Myocardial E/e’ * -0.0064±0.0042 =0.13

Pulse pressure versus

LV mass index * 0.400±0.084 <0.0001

* + SV 0.199±0.069 <0.005

Mean LV wall thickness * 0.00364±0.00074 <0.0001

* + SV 0.00224±0.00070 <0.005

Myocardial s’ * -0.0239±0.012 <0.05

* + SV -0.0262±0.012 <0.05

Myocardial e’ * -0.034±0.015 <0.05

* + SV -0.043±0.015 <0.005

Myocardial E/e’ * 0.0448±0.0159 <0.01

* + SV 0.0531±0.0161 <0.005

_______________________________________________________________

See Figure 1 and Table 1 for other abbreviations. * Adjustments are for age, sex, MAP, regular alcohol intake, regular tobacco intake, BMI, diabetes mellitus, heart rate and hemodynamic factor as indicated.

**Table S3**. Dissociation of relationships between volume (stroke volume [SV])-dependent increases in central arterial pulse pressure (PPc) and left ventricular (LV) structure and function in women of a community with prevalent volume-dependent hypertension (n=524).

Adjustments β-coefficient±SEM p value

____________________________________________________________

Stroke volume versus

LV mass index * 0.099±0.021 <0.0001

Mean LV wall thickness * 0.00129±0.00017 <0.0001

Myocardial s’ * 0.0057±0.0036 =0.12

Myocardial e’ * 0.0053±0.0037 =0.10

Myocardial E/e’ * -0.0061±0.0053 =0.28

Pulse pressure versus

LV mass index * 0.401±0.088 <0.0001

* + SV 0.186±0.081 <0.05

Mean LV wall thickness * 0.00279±0.00073 <0.0005

* + SV 0.00187±0.00070 <0.01

Myocardial s’ * -0.0362±0.0150 <0.05

* + SV -0.0408±0.0153 <0.01

Myocardial e’ * -0.029±0.014 <0.05

* + SV -0.038±0.014 <0.01

Myocardial E/e’ * 0.0530±0.0189 <0.01

* + SV 0.0628±0.0189 <0.005

_______________________________________________________________

See Figure 1 and Table 1 for other abbreviations. * Adjustments are for age, MAP, regular alcohol intake, regular tobacco intake, BMI, diabetes mellitus, treatment for hypertension, heart rate and hemodynamic factor as indicated.

**Table S4**. Dissociation of relationships between volume (stroke volume [SV])-dependent increases in central arterial pulse pressure (PPc) and left ventricular (LV) structure and function in men of a community with prevalent volume-dependent hypertension (n=248).

Adjustments β-coefficient±SEM p value

__________________________________________________________________

Stroke volume versus

LV mass index * 0.150±0.028 <0.0001

Mean LV wall thickness * 0.00193±0.00024 <0.0001

Myocardial s’ * -0.0027±0.0050 =0.59

Myocardial e’ * -0.0016±0.0053 =0.76

Myocardial E/e’ * -0.0026±0.0055 =0.63

Pulse pressure versus

LV mass index * 0.413±0.127 <0.005

* + SV 0.175±0.088 <0.05

Mean LV wall thickness * 0.00362±0.00105 <0.01

* + SV 0.00175±0.00095 =0.07

Myocardial s’ * -0.0234±0.0123 =0.06

* + SV -0.0239±0.0126 =0.06

Myocardial e’ * -0.033±0.017 =0.06

* + SV -0.033±0.017 =0.06

Myocardial E/e’ * 0.0630±0.0197 <0.005

* + SV 0.0636±0.0198 <0.005

_______________________________________________________________

See Figure 1 and Table 1 for other abbreviations. * Adjustments are for age, MAP, regular alcohol intake, regular tobacco intake, BMI, diabetes mellitus, treatment for hypertension, heart rate and hemodynamic factor as indicated.

**Table S5**. Relative impact (standardized β-coefficient) of stroke work (SW) versus indexes of systemic flow (stroke volume [SV] or peak aortic flow [Q] in relationships with left ventricular (LV) mass index (LVMI) or LV mean wall thickness (MWT)(n=772).

β-coefficient±SEM p value

_________________________________________________________________

LVMI versus

SW 0.438±0.041 <0.0001

SV 0.147±0.038 <0.0001

Q -0.052±0.035 =0.14

LV MWT versus

SW 0.055±0.044 =0.21

SV 0.360±0.041 <0.0001

Q -0.005±0.047 =0.91

_________________________________________________________________

All hemodynamic factors were included in the same regression model. Additional adjustments are for age, sex, MAP, regular alcohol intake, regular tobacco intake, BMI, diabetes mellitus, treatment for hypertension, and heart rate.

**Table S6**. Impact of adjustments of determinants of left ventricular afterload on independent relationships between atrial natriuretic peptide concentrations (ANP) and left ventricular function in participants from the community not receiving antihypertensive therapy (n=387).

ANP versus Adjustments β-coefficient±SEM p value

____________________________________________________________

Myocardial s’ * 0.851±0.200 <0.0001

* + PP 0.865±0.202 <0.0001

* + SVR 0.821±0.201 <0.0001

* + Zc 0.808±0.202 <0.0001

* + Pf and Pb 0.886±0.201 <0.0001

* + P_QxZc_ 0.870±0.202 <0.0001

Myocardial e’ * 0.716±0.241 <0.005

* + PP 0.726±0.245 <0.005

* + SVR 0.691±0.243 <0.005

* + Zc 0.698±0.241 <0.005

* + Pf and Pb 0.765±0.239 <0.005

* + P_QxZc_ 0.704±0.242 <0.005

Myocardial E/e’ * -0.183±0.223 =0.41

* + PP -0.241±0.225 =0.29

* + SVR -0.210±0.226 =0.35

* + Zc -0.184±0.223 =0.41

* + Pf and Pb -0.256±0.221 =0.25

* + P_QxZc_  -0.188±0.224 =0.40

____________________________________________________________

See Figure 1 and Table 1 for other abbreviations. * Adjustments are for age, sex, MAP, regular alcohol intake, regular tobacco intake, BMI, diabetes mellitus, heart rate and hemodynamic factors as indicated.

**Table S7**. Impact of adjustments of determinants of left ventricular afterload on independent relationships between atrial natriuretic peptide concentrations (ANP) and left ventricular function in women from a community with prevalent volume-dependent hypertension (n=365).

ANP versus Adjustments β-coefficient±SEM p value

______________________________________________________________

Myocardial s’ * 0.831±0.214 <0.0005

* + PP 0.844±0.214 <0.0001

* + SVR 0.815±0.214 <0.0005

* + Zc 0.789±0.214 <0.0005

* + Pf and Pb 0.818±0.215 <0.0005

* + P_QxZc_ 0.823±0.215 <0.0005

Myocardial e’ * 0.700±0.243 <0.005

* + PP 0.695±0.245 <0.005

* + SVR 0.690±0.244 <0.01

* + Zc 0.687±0.244 <0.01

* + Pf and Pb 0.710±0.240 <0.005

* + P_QxZc_ 0.698±0.243 <0.005

Myocardial E/e’ * -0.291±0.280 =0.30

* + PP -0.349±0.279 =0.21

* + SVR -0.305±0.279 =0.28

* + Zc -0.316±0.280 =0.26

* + Pf and Pb -0.309±0.278 =0.27

* + P_QxZc_  -0.292±0.280 =0.30

_______________________________________________________________

See Figure 1 and Table 1 for other abbreviations. * Adjustments are for age, MAP, regular alcohol intake, regular tobacco intake, BMI, diabetes mellitus, heart rate, antihypertensive therapy and hemodynamic factors as indicated.

**Table S8**. Impact of adjustments of determinants of left ventricular afterload on independent relationships between atrial natriuretic peptide concentrations (ANP) and left ventricular function in men from a community with prevalent volume-dependent hypertension (n=172).

ANP versus Adjustments β-coefficient±SEM p value

____________________________________________________________

Myocardial s’ * 0.709±0.275 <0.05

* + PP 0.730±0.278 <0.01

* + SVR 0.736±0.283 <0.05

* + Zc 0.733±0.283 <0.05

* + Pf and Pb 0.756±0.279 <0.01

* + P_QxZc_ 0.759±0.280 <0.01

Myocardial e’ * 0.743±0.350 <0.05

* + PP 0.783±0.354 <0.05

* + SVR 0.790±0.354 <0.05

* + Zc 0.747±0.351 <0.05

* + Pf and Pb 0.732±0.342 <0.05

* + P_QxZc_ 0.741±0.351 <0.05

Myocardial E/e’ * -0.181±0.320 =0.57

* + PP -0.224±0.331 =0.50

* + SVR -0.235±0.334 =0.48

* + Zc -0.187±0.326 =0.57

* + Pf and Pb -0.208±0.316 =0.51

* + P_QxZc_  -0.201±0.320 =0.53

___________________________________________________________

See Figure 1 and Table 1 for other abbreviations. * Adjustments are for age, MAP, regular alcohol intake, regular tobacco intake, BMI, diabetes mellitus, heart rate, antihypertensive therapy and hemodynamic factors as indicated.

**Table S9**. Relative impact (standardised β-coefficient) of atrial natriuretic peptide (ANP) versus standard risk factors for the detection of left ventricular diastolic dysfunction (LV DD)(86 of 537) in a community with prevalent volume-dependent hypertension.

LV DD versus β-coefficient±SEM p value

_________________________________________________

ANP -0.117±0.041 <0.005

Age 0.145±0.041 <0.01

Central pulse pressure 0.145±0.055 <0.01

Diabetes mellitus 0.095±0.044 <0.05

_________________________________________________

Factors not associated with LV DD, but included in the regression model were sex, BMI, regular smoking, regular alcohol consumption, treatment for hypertension, MAP, and heart rate.
